# The use of prebiotics during the first year of life for atopy prevention and treatment

**DOI:** 10.1002/iid3.8

**Published:** 2013-10-30

**Authors:** Priscilla Negrão de Moura, Nelson Augusto Rosário Filho

**Affiliations:** 1 Nursing School of Ribeirão Preto, University of São Paulo (USP)Ribeirão Preto, São Paulo, Brazil; 2 Head of Pediatric Allergy Service, Clinics Hospital, Federal University of ParanáCuritiba, Parana, Brazil

**Keywords:** Allergy, atopy, children, hypersensitivity, prebiotics, review

## Abstract

The incidence of allergic diseases has increased in recent decades. Therefore, the aim of this systematic review was to assess the efficacy of prebiotics for the prevention and treatment of allergic manifestations in children. We sought to conduct a systematic review of the effectiveness of prebiotics in the prevention and treatment of allergic diseases in children. We searched the MEDLINE, EMBASE, Cochrane Library, LILACS, SciELO, IBECS, Web of Science and Clinical Trials databases as well as Google Scholar and the references of the articles identified. Randomised clinical trials, in which one of the treatments was performed with prebiotics and the control group was treated with placebo, were included in the review. The data selection were performed by two reviewers, and the study quality was evaluated according to the Consolidated Standards of Reporting Trials (CONSORT) items, according to the recommendations for improving the quality of reports of randomised clinical trials. The selected studies showed heterogeneity with regard to the participants, albeit with similar outcomes. The treatment group size ranged from 134 to 259 children, and the studies compared prebiotic to placebo treatment in each group. In general, these articles showed a trend toward less allergic reactions in the groups receiving active therapy with prebiotics. Although there was a trend for reduced allergic symptoms following the administration of prebiotics, there was not sufficient evidence to establish that such treatment is effective for the prevention of allergies in children.

## Introduction

The modern societies of developed countries are associated with a disease profile that differs from that observed decades ago, when infectious diseases prevailed. However, the incidence of allergic diseases has increased in recent decades, and approximately 20% of the Western population suffers from some form of allergic disease, especially those related to food, autoimmune disease or chronic inflammatory disease. The same is also true in developing countries, where these processes may coexist with infectious diseases. This increase in allergic disease appears to result from a shift towards more hygienic habits, which lead to reduced contact between children and microorganisms, as well as familial predisposition and environmental factors. Immunizations and dietary changes also contribute to the development of allergies, and both have significant impacts on the intestinal microbiota [1,2].

The prevalence of atopic dermatitis has increased in recent decades, and one hypothesis to explain this increase is that changes in the pattern of intestinal colonization during childhood have an impact on the immune system.

The oligosaccharides in human milk promote intestinal microflora, predominantly lactobacilli and bifidobacteria, and the mixture of 90% short-chain galactooligosaccharides (GOS) and 10% long-chain fructooligosaccharides (FOS) mimics the prebiotic effect of human milk. GOS and FOS are resistant to digestion and may be detected in the feces of breast-fed infants. Furthermore, infants fed formula supplemented with GOS/FOS possess a microbiota that is similar to those that are breastfed [1,3].

Human milk oligosaccharides also directly interact with immune cells to inhibit the adhesion of pathogens to the intestinal epithelium in addition to their prebiotic effect. The body's greatest mass of lymphoid tissue is located in the digestive tract and is termed the gut-associated lymphoid tissues (GALT), where numerous interactions occur between intestinal bacteria, dendritic cells and epithelial cells. In addition, the microbiota participates in the induction of oral tolerance, a process directed towards specific antigens following their ingestion, which is mediated by Treg cells [4].

However, evidence from randomised trials in the prevention and treatment of atopic dermatitis and food allergies through the use of prebiotics has demonstrated conflicting results. Seven studies on prevention and 12 studies focusing on treatment with prebiotics were identified using the PubMed (Public Medicine), Cochrane (Cochrane Database of Systematic Reviews, CDSR) and EMBASE (Excerpta Medica) databases, and although pro-, pre- and synbiotics have been proposed as potential candidates for the prevention and treatment of atopic dermatitis, the results are not sufficiently conclusive to support the recommendation of their use in this clinical condition [5,6].

Therefore, the aim of this systematic review was to assess the efficacy of prebiotics for the prevention and treatment of allergic manifestations in children.

## Materials and Methods

### Methods

This study is a systematic literature review, characterised as a tool of evidence-based practice, which enables one to summarise and analyse the knowledge produced on the topic examined and thus has a methodological rigor that increases the reliability and depth of the review findings [7].

### Eligibility criteria

Studies that met the following criteria were considered eligible: (1) randomised controlled clinical trials, open-label or blinded; (2) treatment consisting of prebiotics for the prevention and/or treatment of atopy; (3) children 1 year old or younger and (4) data available to measure the treatment effect as a difference in allergic manifestations between groups.

### Data source

Electronic searches for articles published up until September 2012 were conducted in the following databases: MEDLINE (PubMed), EMBASE, Cochrane Library clinical trials registry, LILACS, SciELO, IBECS, Web of Science, Clinical Trials, and Google Scholar. The search for dissertations, theses and conference proceedings was conducted using the Google Scholar search tool. There was no restriction on language or publication year. The return list of each search was copied into a single list of abstracts, removing duplicate entries.

### Search strategy

The search strategy was performed using the following keywords: *hypersensitivity*, *probiotics*, and *child*. The strategy was adapted to each database, when necessary.

### Study selection

The eligibility criteria were applied to all titles and abstracts by two reviewers (PNM, NRF). Cases of disagreement were discussed by the reviewers until they reached a consensus. The references of the selected studies were assessed as a source of new references.

### Data extraction

Data from selected articles were independently extracted by two researchers (PNM, NRF) into a references and notes manager. Cases of disagreement were resolved by consensus. The researchers were not blinded for journal or authors. The study authors were contacted in case of doubt or the absence of specific data.

The following items were collected: sample characteristics, diagnostic measures of atopy, characteristics of the groups compared and aspects of methodological quality. The reduction of allergic symptoms was the primary outcome chosen to test the efficacy of the treatment. Adverse effects of the treatments were analysed as a secondary outcome.

### Quality assessment

The quality of the articles was assessed according to CONSORT [8,9] items, and the articles were classified by reviewers as high or low quality.

### Summary measures

The primary measure of treatment effect was the difference in the percentage of children with allergic symptoms between the groups compared.

### Summary of results

The Preferred Reporting Items for Systematic Reviews and Meta-Analyses (PRISMA) guidelines were followed in the preparation of this systematic review [10].

## Results

**Figure 1:**
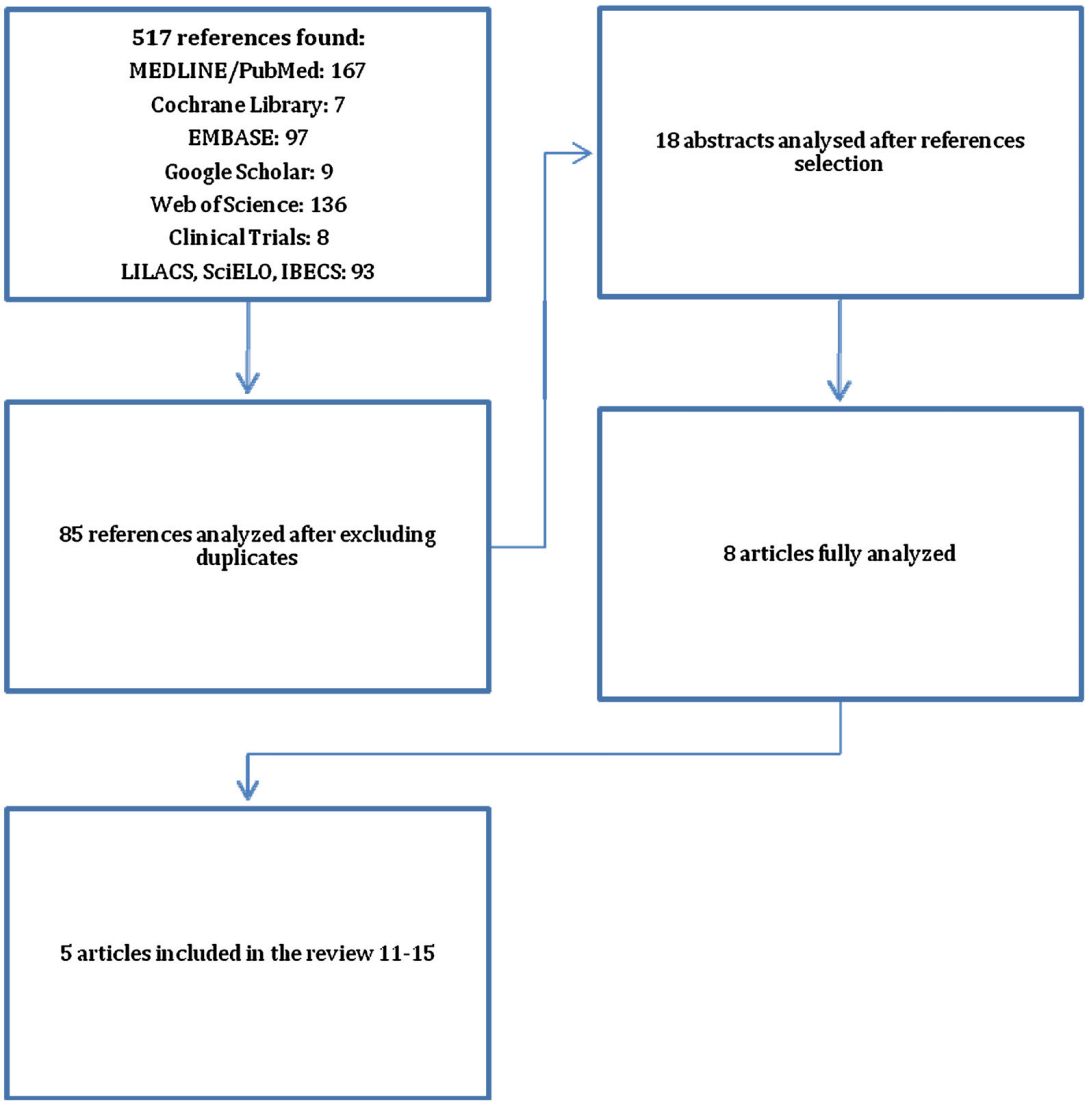
Diagram of the article selection process for meta-analysis.

Eighty-five of a total of 517 references located in nine databases remained for the analysis after excluding duplicate references and performing a new triage. Eighteen of those references were initially included based on the reading of abstracts. Eight articles remained after excluding 10 studies that failed to meet the inclusion criteria. Three more articles were excluded after reading the full articles because they showed a population or intervention that differed from the eligibility criteria. The selection process of the studies is shown in Figure 1.

Five hundred and seventeen articles and eight double-blinded, randomised and placebo-controlled clinical trials with prebiotics and synbiotics were published. Those with allergic diseases as the outcome that were limited to infants or children were selected.

The characteristics of the five clinical trials that met the inclusion criteria [11–15] are outlined in Table1.

**Table 1:** Recently published clinical trials on the effectiveness of prebiotic supplementation for the treatment and/or prevention of allergic diseases

Reference	Type of study	Population	Type of mixture	Dose used	Outcome
Scholtens et al. [11]	Double-blind, randomised, placebo-controlled study	215 healthy infants (187 completed the study) in the first 26 weeks of life	GOS/FOS 9:1	6 g/L	Infant formulas supplemented with prebiotics led to higher concentrations of fecal IgA, suggesting a positive effect on mucosal immunity
Arslanoglu et al. [12]	Double-blind, randomised, placebo-controlled study	152 healthy infants of atopic parents (134 completed the study) received prebiotics or placebo for 6 months and were followed for 2 years	GOS/FOS 9:1	8 g/L	Early dietary supplementation with prebiotics promotes a protective effect against atopic diseases (AD, recurrent bronchospasm and urticaria) and infectious diseases in the first 2 years of life
Moro et al. [13]	Double-blind, randomised, placebo-controlled study	259 infants at risk of atopy were randomly assigned to receive infant formula with and without prebiotics during the first 6 months of life	GOS/FOS 9:1	8 g/L	The group supplemented with prebiotics showed a lower incidence of atopic dermatitis (9.8% vs. 23.1%) and a higher number of bifidogenic bacteria in the gastrointestinal tract
Passeron et al. [14]	Double-blind, randomised, placebo-controlled study	39 children with moderate and severe AD older than 2 years of age used synbiotics or prebiotics alone (control) for 3 months	*Lactobacillus rhamnosus*/GG (LGG), GOS/FOS 9:1	LGG – 1.2 × 10^9^ ufc/g, GOS/FOS	Both synbiotics and prebiotics used alone improved the symptoms of AD in children older than 2 years of age, although two episodes of abdominal pain were noted in the group of synbiotics and one episode was observed in the group of prebiotics
Wu et al. [15]	Double-blind, randomised, placebo-controlled study	54 children from 2 to 14 years of age with moderate to severe AD used synbiotics or prebiotics (control) for 8 weeks	*Lactobacillus salivarius*/FOS, FOS	>2 × 10^9^ ufc/g + FOS, FOS	The combination with synbiotics showed a greater effect in reducing severe AD than did using the prebiotic alone, although two patients in the group of synbiotics initially had diarrhea as a side effect

GOS, galactooligosaccharide; FOS, fructooligosaccharide; AD, atopic dermatitis.

### Characteristics of the studies

The total number of subjects in each study ranged from 134 to 259 infants with prebiotic supplementation only [11–13], whereas the total number of subjects with prebiotic and synbiotic supplementation was 39 and 60 children, respectively [14,15]. Infants, newborns and children 14 years of age or younger were included.

Three studies on prebiotics were included in this review; all used the mixture GOS:FOS (9:1) in infant formulas in nursing infants in the first months of life [11–13] (Table1). The positive effects found were that supplementation changed the intestinal flora, promoting a bifidogenic effect, and supplementation reduced the incidence of allergic diseases (atopic eczema, recurrent bronchospasm, and urticaria) in the first 2 years of life in children at risk for atopy. Two studies on synbiotics whose outcomes were related to allergic diseases were also included. Passeron et al. [14] used GOS:FOS combined with probiotics (LGG) or not in children with moderate and severe atopic dermatitis, and both groups showed improvement of their clinical signs based on severity scores.

Passeron et al. [14] and Wu et al. [15] noted a difference between groups in the onset of mild adverse effects, including diarrhea and abdominal pain, especially in the group supplemented with synbiotics.

Relatively small sample sizes were noticeable in the studies analysed, which are less accurate and consequently have confidence intervals with greater amplitudes.

The selected studies showed heterogeneity regarding the subjects, albeit with similar outcomes. The treatment groups ranged from 39 to 259 children. The studies compared prebiotics and placebo in each group. The synbiotics were considered placebos in two studies to conduct the meta-analysis. In general, the articles reported a trend towards fewer allergic manifestations in the treatment groups with prebiotics. Some adverse effects were reported.

## Discussion

**Figure 2:**
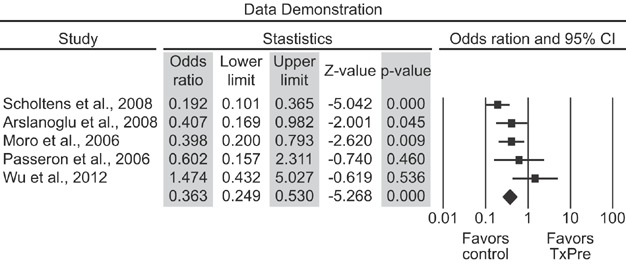
Forest plot depicting a meta-analysis of the effect of prebiotics on allergic manifestations.

The vertical line shows where the odds ratios equal to 1 would be represented, which would indicate the absence of an association between treatment with prebiotics and the occurrence of allergic manifestations. Thus, the odds ratios represented to the left of this vertical line show that allergic manifestations are more likely to occur among controls, and the odds ratios shown to the right of this line demonstrate that allergic manifestations are more likely to occur among those treated with prebiotics. The 95% confidence interval will have a value of 1 if the horizontal line crosses the vertical line, which shows that the effect of using prebiotics on the occurrence of allergic manifestations in that particular study is not statistically significant (the interpretation is similar to that usually performed when the value of *P* is greater than 5%, although it should be noted that this concept of “statistical significance” is always affected by the sample size). Figure 2 shows that the confidence intervals associated with studies 4 and 5 have a value of 1, indicating that their respective odds ratios (OR) are “not statistically significant.” Conversely, studies 4 and 5 noticeably have smaller sample sizes than do the others, suggesting that this “statistical significance” may have not been reached because of the reduced number of subjects in the studies.

Finally, the meta-analytic OR is represented below the set of horizontal lines, with its respective confidence interval (which is also represented by a horizontal line). This result is represented by a rhombus in Figure 2 to differentiate it from the odds ratios associated with each study. Figure 2 shows that the confidence interval for the meta-analytic OR naturally has a smaller amplitude relative to the individual confidence intervals because it gathers data from all of the studies analysed. Figure 2 shows a forest plot for the meta-analysis of the effects of the prebiotics on allergic manifestations. Most odds ratios in this graphic are shown to the left of the dotted vertical line that crosses the scale at one, showing that allergic manifestations have lower incidence rates among subjects who received prebiotics. The meta-analysis Mantel–Haenszel odds ratio has a confidence interval that does not cross the vertical line, which indicates that prebiotics have a statistically significant treatment effect on the reduction of allergic manifestations.

There are essentially two types of regression models used in meta-analysis: fixed effects and random effects models. The random effects models assumes that studies are not homogeneous, with some variation across studies due to differences between their populations and protocols used (for example, dosage or treatment duration).

The progressive speculation on the effectiveness of probiotics and prebiotics resulted from studies that have deepened our knowledge of the immunomodulatory components of breast milk and the related benefits for reducing allergic diseases. Clinical trials sought to evaluate how the management of those components, independently or in combination, either as drugs or as food supplements, could affect atopy-related outcomes [16–18]. In terms of quantity, oligosaccharides are the third greatest component of human milk, following only fat and lactose [19,20]. Recent studies have shown that supplementing infant formula with oligosaccharides leads to long-term benefits in the immune response, including increased production of IgA and a lower incidence of allergic diseases. Similar oligosaccharides may be detected in the feces and urine of breastfed infants, which indicates that these oligosaccharides likely have local and systemic functions [20]. Cytokines derived from T helper cells (Th1 and Th2) and regulatory T cell (Treg) lymphocytes are presumably transported to the systemic circulation via the mesenteric lymph nodes, providing systemic protection effects [21].

This review covers articles related to the topic and published in recent years; the main studies using prebiotics, probiotics, and synbiotics in the prevention and treatment of allergic diseases emerged more recently [21]. Adverse effects of supplementation are rare, although reports of abdominal pain and diarrhea were found when synbiotics were used.

Studies on probiotics with allergic diseases as the outcome, however, are numerous. The vast majority of studies report the role of probiotics in the prevention of allergic diseases but not in their treatment [22]. Therefore, these studies show more robust evidence, especially in the prevention of atopic dermatitis in infants at risk—a result inclusively confirmed by a recent meta-analysis [23]. In turn, the use of prebiotics (GOS:FOS 9:1) during the third trimester of pregnancy leads to an increase in the bifidogenic bacteria in the mothers' gastrointestinal tract, although no such effect was found in the children's flora or caused changes in levels of immunological markers [24]. The recent findings on the importance of intestinal microbiota development in early life on the long-term balance of the immune response significantly increased the interest in the development of research studies to elucidate the mechanisms, components and nutrients involved in that process, highlighting supplementation with prebiotics and probiotics [21,25,26].

However, there are still few studies that investigate a relatively short follow-up period, generally few days or even months. New studies with longer follow-up periods are needed to assess the maintenance of the beneficial effects and safety of using prebiotics. There are still few studies that enable one to consistently assess the beneficial effects of prebiotics and to recommend their use in clinical practice for allergic diseases other than atopic dermatitis.

Meta-analysis can be used to address the effects of clinical therapeutic interventions without a consensus in published studies or in the absence of adequate proof of effectiveness of a specific procedure, provided that certain criteria are met.

This review of studies showed evidence of the benefits of early supplementation with prebiotics in the prevention of atopic dermatitis in infants at high risk for allergies. However, there is still little evidence available, and the results regarding prebiotic adjuvant treatment for moderate to severe atopic dermatitis mediated by IgE are controversial. Studies including a more prolonged period of observation for supplemented subjects and an evaluation of product safety and long-term effects are also needed.

Although we found a tendency towards reduced allergic reactions upon supplementation with prebiotics, there was not sufficient evidence to assert that this treatment is effective for preventing allergies in children.

In conclusion, supplementation with prebiotics mimicking breast milk may reduce the frequency of infections and atopy in healthy infants. However, the long-term benefits of prebiotics for the developing immune system remain to be further elucidated [3,4,6,7,27].
